# Phytohormone Involvement in the *Ustilago maydis– Zea mays* Pathosystem: Relationships between Abscisic Acid and Cytokinin Levels and Strain Virulence in Infected Cob Tissue

**DOI:** 10.1371/journal.pone.0130945

**Published:** 2015-06-24

**Authors:** Erin N. Morrison, R. J. Neil Emery, Barry J. Saville

**Affiliations:** 1 Environmental and Life Sciences Graduate Program, Trent University, Peterborough, Ontario, Canada; 2 Biology Department, Trent University, Peterborough, Ontario, Canada; 3 Forensic Science Program, Trent University, Peterborough, Ontario, Canada; Henan Agricultural Univerisity, CHINA

## Abstract

*Ustilago maydis* is the causative agent of common smut of corn. Early studies noted its ability to synthesize phytohormones and, more recently these growth promoting substances were confirmed as cytokinins (CKs). Cytokinins comprise a group of phytohormones commonly associated with actively dividing tissues. Lab analyses identified variation in virulence between *U*. *maydis* dikaryon and solopathogen infections of corn cob tissue. Samples from infected cob tissue were taken at sequential time points post infection and biochemical profiling was performed using high performance liquid chromatography-electrospray ionization tandem mass spectrometry (HPLC-ESI MS/MS). This hormone profiling revealed that there were altered levels of ABA and major CKs, with a marked reduction in CK glucosides, increases in methylthiol CKs and a particularly dramatic increase in *cis*Z CK forms, in *U*. *maydis* infected tissue. These changes were more pronounced in the more virulent dikaryon relative to the solopathogenic strain suggesting a role for cytokinins in moderating virulence during biotrophic infection. These findings highlight the fact that *U*. *maydis* does not simply mimic a fertilized seed but instead reprograms the host tissue. Results underscore the suitability of the *Ustilago maydis– Zea mays* model as a basis for investigating the control of phytohormone dynamics during biotrophic infection of plants.

## Introduction

A key strategy for successful pathogen establishment often involves hijacking an already existing plant network. Manipulation of host metabolism through the production of phytohormones by plant pathogens, or the activation of host phytohormone biosynthesis, is often used as a component of establishing pathogen growth within the host [1,2].

Phytohormone manipulation has often been associated with the infection strategy of biotrophic fungi through; nutrient diversion, suppression of plant defense responses, and increased host susceptibility, all of which have been mimicked through the exogenous application of the phytohormones cytokinins (CK) or abscisic acid (ABA) [3–5].

The phytohormone group cytokinins are N^6^ substituted adenine derivatives which are important in a number of developmental processes within the plant including cell division and differentiation [6]; abscisic acid, is often referred to as the plant stress hormone, and is involved in plant development as well as adaptation to various environmental biotic and abiotic stressors [5,7,8].

While phytohormone production is often associated with biotrophic fungi, the key questions remain as to why non-plant associated microbes produce phytohormones? And, what is the mechanism by which microbial phytohormone production provides a pathogenic advantage in a host-pathogen interaction? [4](reviewed in [9], [10], reviewed in [11], [12–14]). Here we examine the latter of the two questions.

Studies examining the infection process of the basidiomycete corn smut fungus *Ustilago maydis* have suggested that phytohormone manipulation, by this fungus, may play a role in host infection [15,16]. Infection of corn by the *U*. *maydis* pathogenic dikaryon stimulates uncoordinated cellular division, resulting in tumour formation on aerial portions of the plant including the cob [17,18]. Within these tumours black, diploid teliospores are produced which act as the dispersal agent for the fungus [18,19]. *U*. *maydis* infection of *Zea mays* results in reduced photosynthetic rate, maintenance of nutrient sinks [20], elevated levels of total soluble sugars, and increased nitrogen accumulation in infected tumours [21]. These features are common among biotrophic fungal infections and have been mimicked through the application of exogenous CKs [1]. However, the link to CKs during the *U*. *maydis*- *Zea mays* interaction has not been thoroughly examined, although previous studies have shown that cultured *U*. *maydis* (sporidia and dikaryon) were capable of producing CKs and ABA, and that specific CK forms varied during infection of maize seedlings [22,23], none have examined the hallmark of this disease, cob tumour formation.

Information for *U*. *maydis* phytohormone manipulation and pathogenesis has mostly been done in corn seedlings however, seedling infections elicit symptoms that are distinctly different from those seen in cob tissue and, therefore, likely result in different CK profiles. *U*. *maydis* pathogenesis assays are often carried out using the *U*. *maydis* dikaryon, which results from the fusion of two compatible haploid sporidia, or using a genetically engineered haploid solopathogen [18,19]. In lab observations it was noted that infection of corn seedlings and cob tissue by these two strains resulted in different rates of disease development and severity of disease symptoms, with the dikaryon being more virulent (S1 Fig). With this in mind we sought to examine the changes in ABA and CK profiles between mock-infected and *U*. *maydis* infected cob tissue at specific time points post infection, and determine if *U*. *maydis* strains (dikaryon vs. solopathogen) with contrasting virulence will result in different ABA and CK profiles during infection. Samples, from infected cob tissue, were taken at various time points post infection and biochemical profiling conducted using high performance liquid chromatography-electrospray ionization tandem mass spectrometry (HPLC-ESI MS/MS). The use of HPLC-ESI MS/MS in this study permits the detection and separation of ABA and CK analytes from the same tissue sample [14]. In this study, it was observed that ABA levels were elevated in infected tissue. CK levels specific to the free base, riboside, nucleotide (FBRNT) group of CKs increased dramatically in *U*. *maydis* infected tissue relative to the mock-infected control, and further differences were found when comparing the dikaryon and solopathogen infections. This comparison of ABA and CK profiles between different *U*. *maydis* strain infections will provide new insight into virulence factors experienced during infection.

## Materials and Methods

### *Ustilago maydis* strains and growth conditions

*Ustilago maydis* strains used in the experiments include: FB1 (*a1b1*) and FB2 (*a2b2*), provided by Flora Banuett [19], and the solopathogenic haploid strain SG200 (*a1 mfa2 bE1bW2*); a FB1 derived strain engineered to grow filamentously and cause disease without a mating partner, obtained from Jörg Kämper (Karlsruhe Institute of Technology, Karlsruhe, Germany; [18,24]). Budding cultures were grown on solid YEPS medium (1% w/v yeast extract, 2% w/v peptone, 2% w/v sucrose) containing agar for 3–4 days at 28 C; for SG200, solid medium was supplemented with 20 μg mL^-1^ phleomycin (InvivoGen). Single colonies were inoculated into liquid YEPS medium and grown overnight (28 C, 250 rpm). Two hundred μL of overnight culture was inoculated into 200 mL of liquid YEPS medium and grown overnight (28 C, 250 rpm). Cultures were diluted to a final OD_600_ of 1 using sterile dH_2_O. For compatible haploids (FB1 x FB2) equal volumes of diluted culture were combined. For solopathogenic (SG200) injections, cultures were diluted to a final OD_600_ of 1.

### Plant growth conditions, tissue injection and sampling

*Zea mays* L. ‘Golden Bantam’ (Ontario Seed Company, Canada) seeds were planted (16 per 38 cm pot, reduced to 8 plants per pot) in Sunshine Professional Growing Mix (Mix#1, Sungro Horticulture Canada). Two pots with a total of 10 cobs were used for the mock-infected treatment, three pots with a total of 14 cobs were used for the dikaryon (FB1 x FB2) treatment and four pots with a total of 13 cobs were used for the solopathogen (SG200) treatment. Germination and growth occurred under 16 h light, 60–80% RH, 24–27 C, 8 h dark, 60–80% RH, at 19–22 C (Aurora Greenhouse, Conviron, Canada). Corn plants were detasseled to prevent pollination of ovules and increase the likelihood of cob infection [25,26]. Cobs were injected, as described in Morrison et al. [27]. Briefly, *U*. *maydis* injection of cobs was carried out using 6 mL of culture (FB1 x FB2 or SG200), which was diluted to an OD_600_ of 1 and injected down the silk shaft of cobs using a 10 mL syringe and 18 gauge needle. If strong back pressure occurred (suggesting insertion into the cob) the needle was drawn back or reinserted through the husk at a different location. Mock-infections (controls) were injected in the same manner using diluted YEPS (50%v/v sdH_2_O).

### Time course tissue sampling

**Fig 1:**
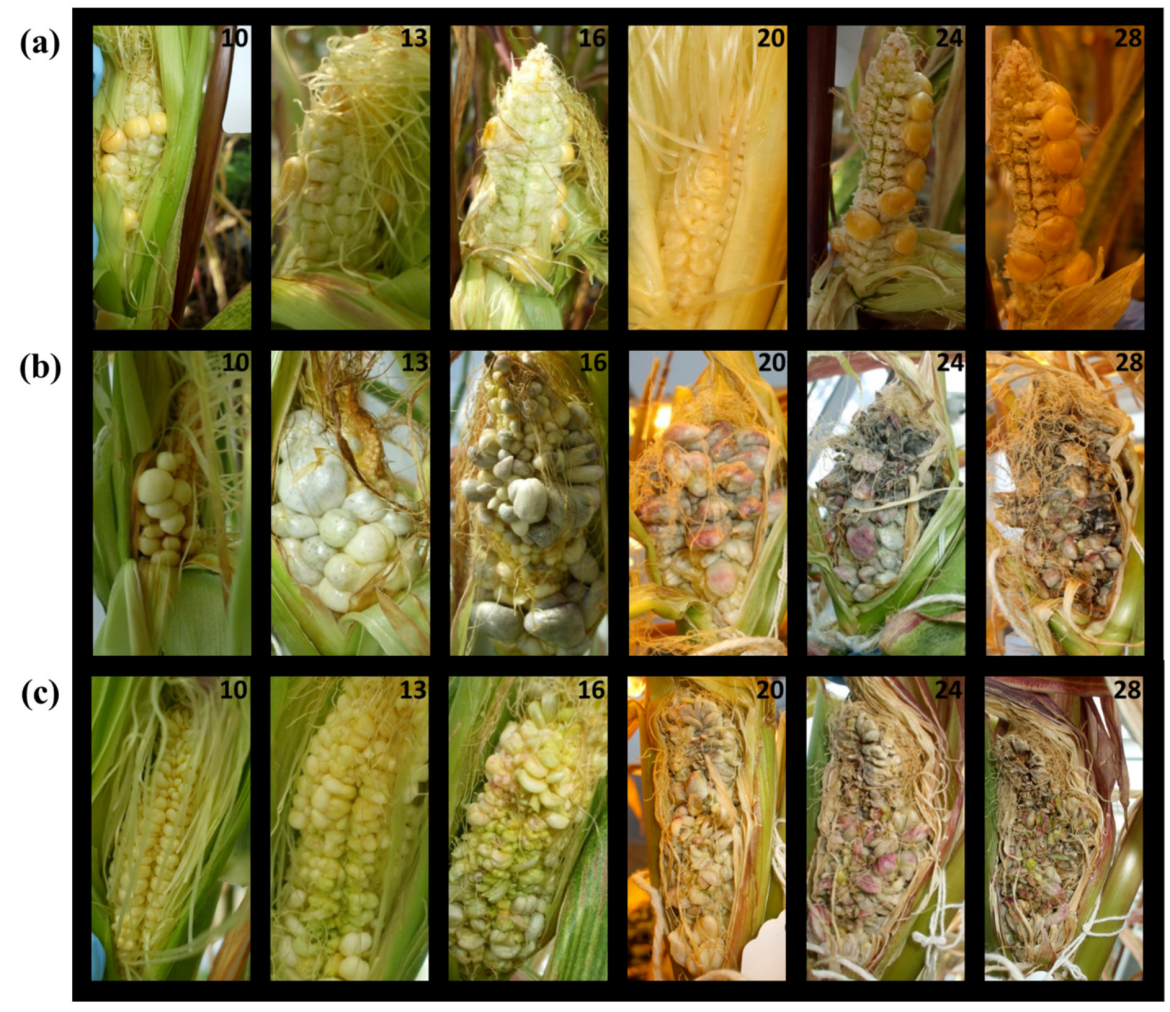
Representative time course of disease progression for *Zea mays- U*. *maydis* cob assay. (a) Mock-infected controls (b) Dikaryon (FB1 x FB2) infected (c) Solopathogen (SG200) infected. Days post infection (dpi) appear in the top right-hand corners of individual photographs.

Disease progression was monitored and tissue samples were taken at 10, 13, 16, 20, 24 and 28 days post infection (dpi). Fig 1 shows the progression of disease symptoms for the mock-infected control, dikaryon (FB1 x FB2), and solopathogen (SG200). Sample days were based on in-lab observations of disease progression and documented stages in Pataky and Snetselaar [28]. Sampling of infected cobs was heavily reliant on the presence of infected tissue and absence of secondary infection. Tissue was selected based on disease progression at a given time point, therefore sampling was not limited to a single pot per treatment. Cobs were opened in-line with husk growth and examined for signs of infection. Infected sites were excised using a razor blade, weighed, frozen in liquid nitrogen and stored at -80 C until phytohormone extraction could take place. Following sampling, cobs were covered again using existing husk material. While infection rates can increase with silk-channel injection, not all cobs will develop disease symptoms [25]. To gain adequate sample numbers, infected cobs were sampled throughout the progression of the disease even if samples were previously taken from the same cob; however, tissue was not sampled from a previously excised region of the cob even if re-growth was visible. *U*. *maydis* infected tissue from early time points required pooling of small tumours to reach the tissue mass required for hormone extraction. Later time points, with larger tumours, allowed single tumours to represent a single sample. Each tumour in later time points was considered an individual sample even if it was taken from the same cob. Table 1 presents a summary of the number of cobs sampled and the total number of samples collected at each time point. Unfertilized ovules from mock-infected controls were often pooled to represent one sample. For each time point, an attempt was made to examine a previously unopened cob, when available.

**Table 1:** Tissue sampling for phytohormone analysis.

Days Post Infection	Injected strain	Number of Cobs sampled	Newly opened Cobs	Total samples (n)
10	Control	2	2	2
	FB1xFB2	2	2	2
	SG200	2	2	2
13	Control	3	1	3
	FB1xFB2	3	1	3
	SG200	3	1	3
16	Control	4	2	4
	FB1xFB2	4	1	4
	SG200	4	2	4
20	Control	6	1	6
	FB1xFB2	5	1	5
	SG200	4	0	5
24	Control	3	0	3
	FB1xFB2	6	1	11
	SG200	4	0	5
28	Control	4	1	4
	FB1xFB2	8	2	15
	SG200	4	0	6

### CK and ABA extraction and purification

Hormone extraction and purification were carried out as described in Ross et al. [29] for ABA and in Dobrev and Kaminek [30], as modified in Morrison et al. [14] for CKs. Briefly, frozen tissue was reweighed and internal standards added during the first extraction step to enable endogenous hormone quantification through the isotope dilution technique [31]. Internal standards included 146.9 ng of labeled ABA (^2^H_4_ABA) (PBI, Saskatoon), and 10 ng of the following CK’s: ^2^H_7_BA, ^2^H_7_BAR, ^2^H_5_ZOG, ^2^H_7_DHZOG, ^2^H_5_ZROG, ^2^H_7_DHZROG, ^2^H_6_iP7G, ^2^H_5_Z9G, ^2^H_5_MeSZ, ^2^H_6_MeSiP, ^2^H_5_MeZR, ^2^H_6_MeSiPR, ^2^H_6_iPR, ^2^H_3_DHZR, ^2^H_6_iP, ^2^H_3_DHZ, ^2^H_6_iPRMP, and ^2^H_6_DHZRMP (OlchemIm Ltd., Olomouc, CZ). Similarity in retention time allowed the use of labeled ^2^H_3_DHZR, ^2^H_3_DHZ, and ^2^H_6_DHZRMP for the quantification of *trans*Z, *trans*ZR, *trans*ZRP, and *cis*Z isomers. Since deuterated standards were not commercially available for *cis*-CKs, levels of these compounds were quantified using the recovery of the corresponding DHZ-CK deuterated standard. Frozen tissue was homogenized in pre-cooled (-20 C) modified Bieleski #2 extraction buffer (Methanol: Water: Formic Acid; CH_3_OH:H_2_O:HCO_2_H (15:4:1, v/v/v)) using a ball mill grinder and zirconium oxide grinding beads (Comeau Technique Ltd., Vaudreuil-Dorion, Canada; 25 mZ, 5 minutes, 4 C) for tissue ~0.25 g or less. Stainless steel grinding cylinders were used for tissue greater than ~0.25 g (25 mZ, 30 seconds, 4 C, Retsch MM300). Following tissue homogenization samples were sonicated, vortexed and allowed to extract passively overnight at -20 C for approximately 12 hrs. Following overnight extraction, samples were centrifuged at 8400 x *g* for 10 minutes (Sorvall ST 16 Centrifuge or Fisher Scientific Centrifuge at maximum speed) and the supernatant collected. Pellets were re-extracted and allowed to extract passively in modified Bieleski #2 extraction buffer for 30 minutes at -20 C. Samples were centrifuged as above and pooled supernatants were dried in a speed vacuum concentrator at ambient temperature (Savant SPD111V, UVS400, Thermo Fisher Scientific, Waltham, MA). Dried samples were stored at -20 C until use. Dried supernatant residues were reconstituted in 1 mL of 1M HCO_2_H, to allow for complete protonation of CKs, and subjected to solid phase extraction (SPE) on a mixed mode, reverse-phase/ cation-exchange cartridge (Oasis MCX 6 cc; Waters, Mississauga, Canada). Cartridges were activated with 5 mL CH_3_OH and equilibrated with 5 mL 1M HCO_2_H. Following equilibration the sample was loaded and washed with 5 mL 1M HCO_2_H. ABA was eluted first using 5 mL CH_3_OH. CKs were eluted based on their chemical properties, with CK nucleotide forms eluted second, using 5 mL 0.35 M ammonium hydroxide (NH_4_OH) followed by riboside, free base, methylthiol, and glucoside CK forms eluted using 5 mL 0.35 M NH_4_OH in 60% CH_3_OH. Collected fractions were evaporated to dryness and stored at -20 C.

CK nucleotides were reconstituted in 1 mL 0.1 M ethanolamine-HCl (pH 10.4) and dephosphorylated to form ribosides using 3.4 units of bacterial alkaline phosphatase (Sigma, Oakville, Canada) for 12 hours at 37 C. Resulting CK ribosides were evaporated to dryness in a speed vacuum concentrator at ambient temperature. Due to the need for nucleotide to riboside conversion for detection purposes, resultant nucleotide data potentially reflects pooled contribution of mono, di- or tri- phosphates as the isopentenyl or hydroxylated moiety can be transferred to an AMP, ADP or ATP [32] this is represented in the current study by using iPRP, DHZRP, *trans*ZRP and *cis*ZRP to represent the respective pooled nucleotide data for that particular analyte. Dephosphorylated nucleotides were reconstituted in 1.5 mL Milli-Q H_2_O and further purified using a reversed-phase C_18_ SPE column (Oasis C_18_ 3 cc; Waters, Mississauga, Canada). Column activation and equilibration were carried out using 3 mL CH_3_OH and 6 mL Milli-Q H_2_O, respectively. Samples were loaded and allowed to pass through the column under gravity. The sorbent bed was washed with 3 mL Milli-Q H_2_O and samples were eluted using 1.5 mL CH_3_OH. Samples were dried in a speed vacuum concentrator and stored at -20 C until analysis.

Purified fractions of ABA, CK nucleotides, CK ribosides/ free base/ methylthiol/ glucosides were reconstituted in 1.5 mL of initial HPLC mobile phase conditions (95:5 H_2_O: CH_3_OH with 0.08% acetic acid (CH_3_CO_2_H)) for ABA and (95:5 H_2_O: Acetonitrile (C_2_H_3_N) with 0.08% CH_3_CO_2_H) for CKs. Samples were transferred to glass auto- sampler vials and stored at 4 C until analysis.

HPLC-ESI MS/MS methods with multiple reaction monitoring (MRM) channels, specific for each analyte, were carried out as described in Ross et al. [29] and Farrow and Emery [33]. Detection limits were as listed in Farrow and Emery [33]. Samples were analyzed and quantified by HPLC-ESI MS/MS (Agilent 1100 series HPLC connected to a Sciex Applied Biosystem 5500 API mass spectrometer) with a turbo V-spray ionization source. A 20 μL sample was injected onto a Luna C_18_ reverse-phase HPLC column (3 μm, 150 x 2.0 mm; Phenomenex, Torrance, CA, U.S.A.); all ABA samples were analyzed in negative-ion (ESI-) mode and all CK samples were analyzed in positive-ion (ESI+) mode. ABA was eluted using component A: H_2_O with 0.08% CH_3_CO_2_H and component B: CH_3_OH with 0.08% CH_3_CO_2_H, at a flow rate of 0.2 mL minute^-1^. CKs were eluted using component A: H_2_O with 0.08% CH_3_CO_2_H and component B: C_2_H_3_N with 0.08% CH_3_CO_2_H, at a flow rate of 0.2 mL minute^-1^. Initial conditions for the ABA fraction were 50% B changing on a linear gradient to 80% B over 8 minutes. This ratio was then held constant for 2 minutes before returning to starting conditions and equilibrating for 8 minutes. The CK fractions were eluted using a multistep gradient. Starting conditions were 5% B increasing linearly to 95% B over 17 minutes. 95% B was held constant for 5 minutes before returning to starting conditions for 18 minutes.

### Data analysis

Data sets were analyzed using Analyst (v. 1.5) software (AB SCIEX, Concord, Canada). ABA and CKs were identified based on their MRM channels and retention times. Analyte concentrations were determined using isotope dilution analysis based on direct comparison of the endogenous analyte peak area to that of the recovered internal standard [31]. Final hormone concentrations were normalized to the initial fresh weight of the sample. Statistical analysis was carried out using an analysis of variance (ANOVA) with the Tukey-Kramer post-hoc test, which takes unequal sample size into account. Significant differences refer to a *p*-value of <0.05. Where appropriate Student *t*-tests were also conducted (two tailed, assuming unequal variance). In the case of ABA analysis, data points were subjected to the Grubb’s test for outlier detection.

## Results

### Disease progression and tissue sampling

Disease symptoms were monitored and recorded over the course of 28 days. Multiple tissue samples were taken at each time point from the mock-infected (control), the dikaryon and the solopathogen injections. While effort was taken to prevent kernel pollination, in some cases cobs had a mix of fertilized and unfertilized kernels. Snetselaar et al. [25] noted that kernel pollination was not necessary for *U*. *maydis* infection and may actually interfere with fungal development. An effort was made to collect only unfertilized tissue from control cobs, as this was thought to be more representative of the tissue that would be infected by *U*. *maydis*. During the early stages of disease, small, white, disorganized galls were visible (day 10) (Fig 1). Gall enlargement had begun by day 13 and in most cases by day 16 the tumours took on a grey appearance. In some cases, exposed *U*. *maydis* tumours also became green or purple in appearance. By day 20, enlarged tumour tissue was darkened by the development/maturation of teliospores within the tissue. By days 24–28 most tumour tissue had begun to dehydrate. All opened or harvestable cobs were tracked to evaluate the progression of disease. Samples selected for hormone analysis were those in which cob tissue developed disease symptoms. Because of the nature of the experiment, which included destructive and repeated sampling, any cob with secondary infection was discarded. Tissue sample numbers ranged from 2–15, and were determined by the day during which samples were taken, the treatment type and the presence of diseased tissue. During early time points, infection differences were qualitatively observed between the dikaryon and solopathogen strains. Dikaryon infected tissue resulted in tumours that were larger and more bulbous than those from the solopathogen treatment (Fig 1, day 10–16). Infection percentages were determined for the dikaryon and solopathogen treatments, and these were based on whether an individual cob contained tumours that could be harvested for hormone analysis. The dikaryon had 57% harvestable cobs whereas the solopathogen had 38%.

### Abscisic acid

**Fig 2:**
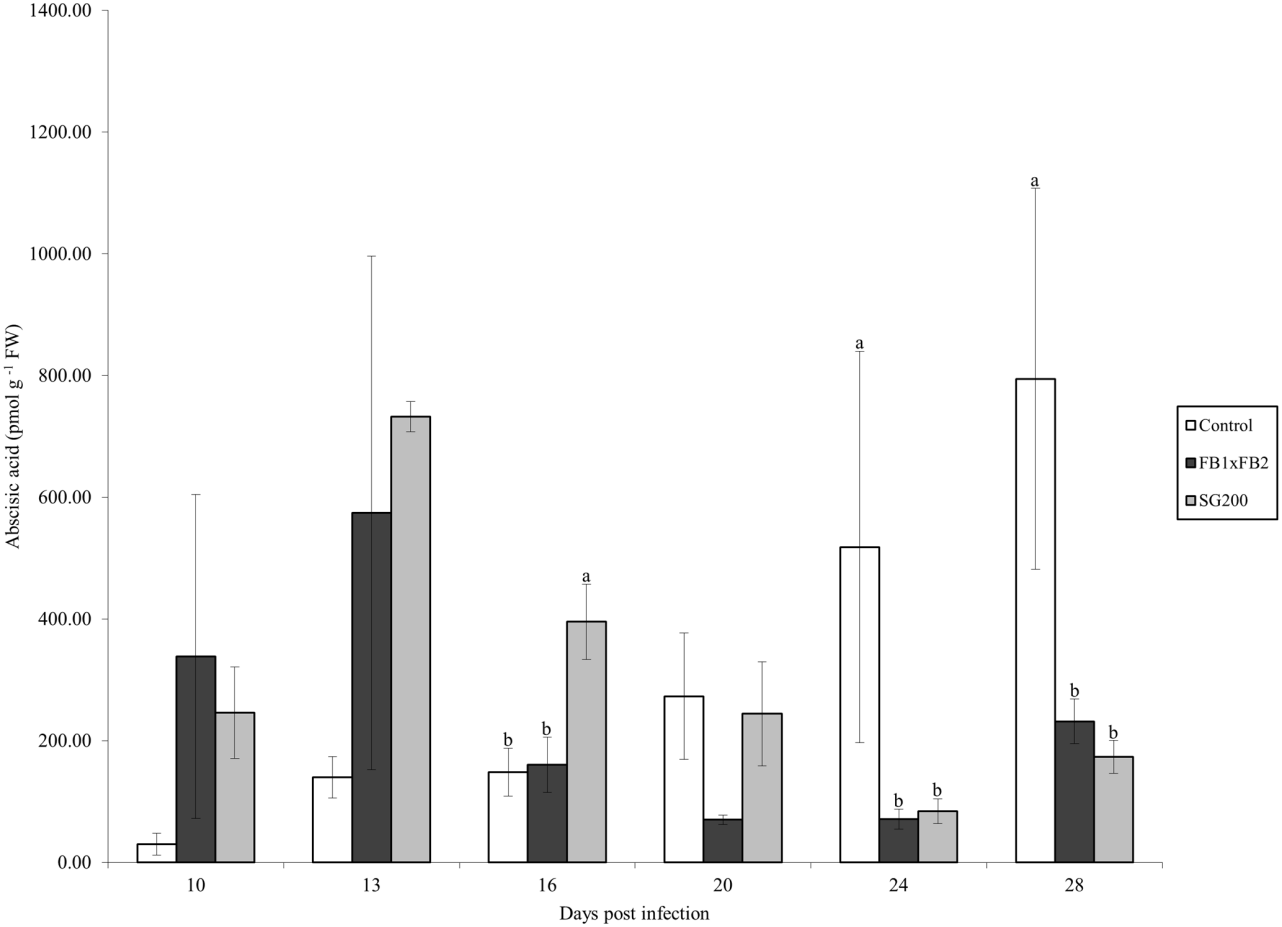
Abscisic acid concentration (pmol g ^-1^ FW) for control, dikaryon and solopathogen injected cob tissue at specific days post infection (dpi). n = 2–15, error bars represent standard error (SE). Letters denote significant differences within time points (ANOVA and Tukey-Kramer post hoc analysis, *p*<0.05).

Tissue samples were extracted and analyzed for the presence of ABA by HPLC-ESI (-) MS/MS. ABA was present in all treatments at all time points. The control treatment ranged from 29.84 pmol g^-1^ FW at day 10 to 794.62 pmol g^-1^ FW at day 28 (Fig 2). The dikaryon treatment reached its highest level of ABA content at day 13 (574.23 pmol g^-1^ FW), which was greatly reduced by day 16 with subsequent days ranging from 70.2 pmol g^-1^ FW to 231.84 pmol g^-1^ FW (days 20 and 28 respectively). The solopathogenic treatment showed an increase in ABA levels at day 13 (732.13 pmol g^-1^ FW), followed by a gradual decrease in total ABA (Fig 2). Significant differences were detected between the solopathogen and other treatments at day 16 (Tukey Kramer *p*<0.05) and between the control tissue and infected tissue at days 24 and 28 (Tukey Kramer *p*<0.05).

### Cytokinins

**Fig 3:**
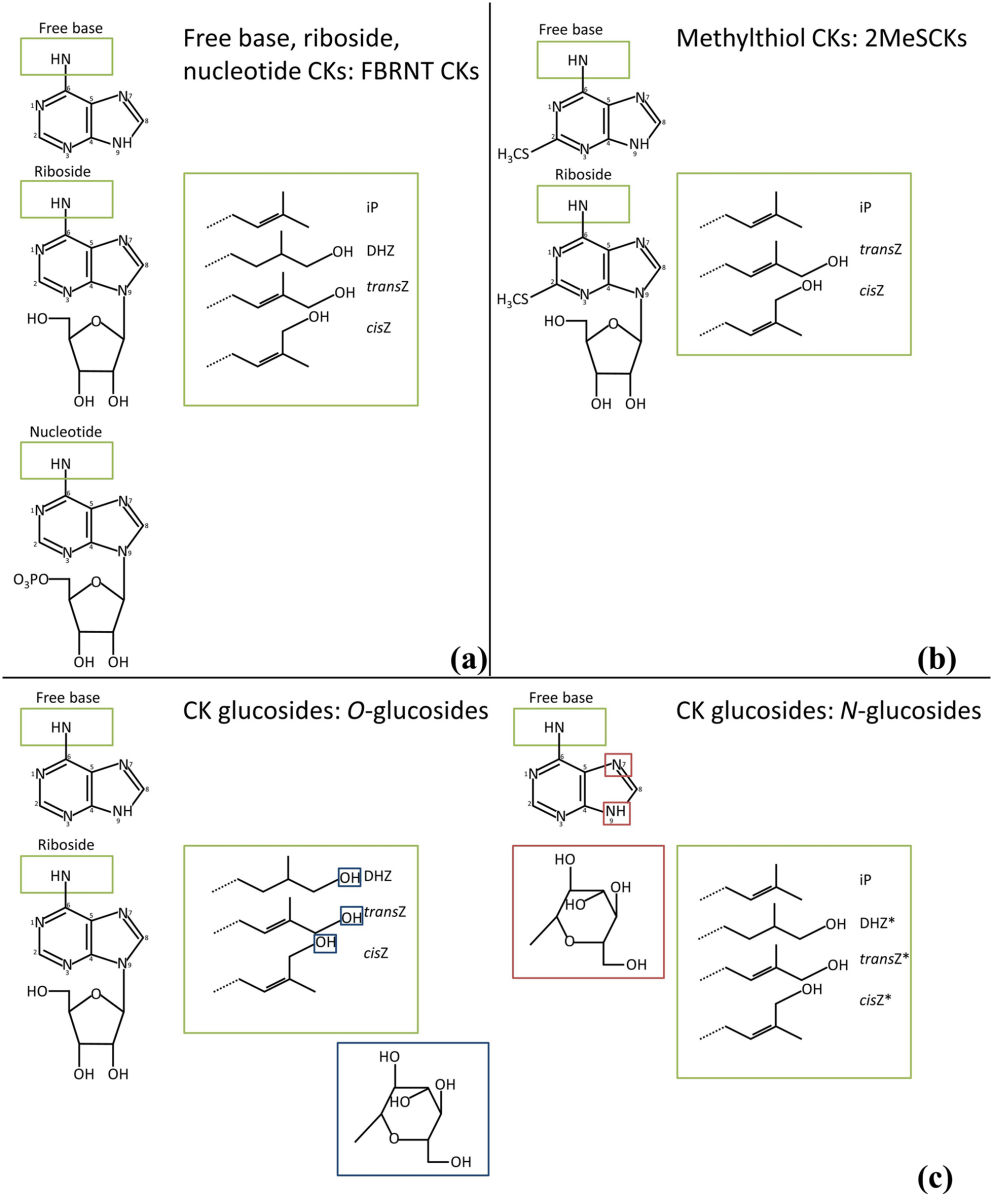
Representative CK structures for analytes detected/scanned for, in the *Zea mays- U*. *maydis* cob assay time course. (a) Free base, riboside, nucleotide CKs (b) Methylthiol CKs (c) CK glucosides including *O*-glucosides (left) and *N*-glucosides (right). Green boxes indicate addition of side chains; large green boxes show the different side chains. Blue boxes indicate the structure of glucose and location of glucosylation for *O*-glucosides. Red boxes indicate the structure of glucose and location of glucosylation for *N*-glucosides. Adapted from: Esberg et al. [70], Sakakibara [34], Frébort et al. [12]. *The 7G form of these analytes was not scanned for.

Total CKs, comprised of individual CK types and forms, were extracted from collected tissue and quantified. CKs were grouped based on their known function or structure into one of three categories: CK glucosides, methylthiol (2MeS) CKs, or freebase, riboside and nucleotide (FBRNT) CKs. The structures for these grouped CKs are shown in Fig 3. Those CKs shown in Fig 3 represent all compounds scanned for on the HPLC-ESI MS/MS (excluding aromatic CKs) including those that were undetected. CK glucosides are considered inactive CK forms [34], and were grouped to include the following analytes: DHZOG, DHZROG, DHZ9G, *trans*ZOG, *trans*ZROG, *trans*Z9G, *cis*ZOG, *cis*ZROG, and *cis*Z9G (iP9G and 7Gs were not detected in this study). Methylthiol CKs are modified at C2 with the addition of a methylthiol group [34] (Fig 3b) and, for this study 2MeSCKs included the following analytes: 2MeZR, 2MeSZ, 2MeSiPR, and 2MeSiP. FBRNT CKs represent the putatively active CK forms and their immediate precursors. In this study, the FBRNT CK grouping included the following analytes: iP, iPR, iPRP, DHZ, DHZR, DHZRP, *trans*Z, *trans*ZR, *trans*ZRP, *cis*Z, *cis*ZR, and *cis*ZRP. Analyte concentrations are found in Tables 2, 3 and 4 for the mock-infected control, dikaryon and solopathogen infected tissues. No aromatic CKs were detected in any tissue type and were therefore not listed in Tables 2, 3 and 4.

**Table 2:** Cytokinin concentrations (pmol g ^-1^ FW) in mock-infected *Z*. *mays* cob tissue, during the *U*. *maydis*- *Z*. *mays* infection time course.

	Mock-infected control					
dpi	10	13	16	20	24	28
n	2	3	4	6	3	4
**Free base**						
iP						
DHZ						
*trans*Z						
*cis*Z						
**Riboside**						
iPR						
DHZR	0.894±0.178	1.316±0.473	1.298±0.347	0.232±0.232		
*trans*ZR						0.766±0.591
*cis*ZR		0.781±0.399	0.901±0.901	0.435±0.435		6.643±1.916
**Nucleotide**						
iPRP						
DHZRP						
*trans*ZRP	11.637±11.637	2.232±1.844	3.360±1.145	2.196±1.693	5.063±2.540	7.323±4.438
*cis*ZRP	13.408±6.592	8.541±3.200	8.408±1.842	5.387±1.561	5.196±1.007	7.287±1.762
**Glucoside**						
iP7G						
iP9G						
DHZOG	33.433±0.599	49.700±8.251	39.251±6.734	87.827±19.847	56.571±11.410	186.302±58.786
DHZROG	10.181±10.181	130.352±50.510	53.194±25.736	146.892±68.871	10.808±10.808	554.287±354.411
DHZ9G	6.251±4.365	9.333±3.162	8.201±2.958	13.351±3.831	7.499±2.585	23.176±8.332
*trans*ZOG				1.289±1.289	3.364±1.115	1.280±0.918
*trans*ZROG						
*trans*Z9G	102.735±35.905	356.656±216.054	242.197±75.138	353.577±71.406	181.965±64.293	407.302±198.627
*cis*ZOG					5.407±5.407	2.617±1.660
*cis*ZROG	15.978±8.585	112.918±47.038	93.553±46.092	108.389±49.763	23.611±10.991	91.305±53.553
*cis*Z9G	151.646±47.509	330.692±60.193	405.499±123.200	322.048±42.930	352.297±29.230	504.984±65.255
**Methylthiol**						
2MeSZR						
2MeSZ						
2MeSiPR						
2MeSiP						

Values are means ± standard error (SE) (n = 2–15) at specific days post infection (dpi). Empty cells indicate values of zero for those analytes. In cases where an analyte was detected in only one sample the presented mean concentration is equivalent to the SE.

**Table 3:** Cytokinin concentrations (pmol g ^-1^ FW) in *U*. *maydis* dikaryon infected *Z*. *mays* cob tissue, during the *U*. *maydis*- *Z*. *mays* infection time course.

	Dikaryon (FB1xFB2)					
dpi	10	13	16	20	24	28
n	2	3	4	5	11	15
**Free base**						
iP				0.725±0.725	3.867±1.335	1.410±0.969
DHZ						
*trans*Z			2.845±1.760	8.447±2.166	12.101±2.955	20.164±5.771
*cis*Z			1.023±0.608		0.196±0.196	1.663±0.505
**Riboside**						
iPR	3.540±1.377	2.260±0.807	4.748±1.557	7.066±1.429	12.092±3.084	20.273±3.914
DHZR	0.993±0.384	0.650±0.259	0.178±0.071		0.359±0.163	0.680±0.366
*trans*ZR	2.545±2.545	0.778±0.091	0.750±0.204	0.404±0.118	1.008±0.218	2.007±0.383
*cis*ZR	3.895±2.062	5.090±0.829	13.081±3.806	37.268±5.463	69.589±17.792	108.695±25.004
**Nucleotide**						
iPRP	7.131±2.470	2.837±1.439	12.612±3.654	12.297±2.722	13.665±2.258	15.622±4.402
DHZRP						
*trans*ZRP	25.780±22.862	5.323±0.841	4.010±1.155	1.566±0.309	1.533±0.256	2.112±0.444
*cis*ZRP	9.673±1.850	31.596±10.957	91.997±21.851	140.087±24.705	178.246±26.170	203.240±40.095
**Glucoside**						
iP7G						
iP9G						
DHZOG					0.251±0.251	0.213±0.213
DHZROG	24.643±	32.723±3.358	32.638±2.931	23.804±5.304	19.218±2.806	27.221±4.876
DHZ9G						
*trans*ZOG					0.016±0.016	
*trans*ZROG						0.150±0.150
*trans*Z9G	3.087±3.087	3.778±0.539	4.651±0.921	5.760±1.338	6.370±1.172	13.909±3.055
*cis*ZOG						
*cis*ZROG	145.494±81.046	152.440±33.993	171.386±32.640	122.555±20.715	121.681±24.195	167.706±29.310
*cis*Z9G	59.823±45.756	34.579±4.953	39.088±12.088	57.224±7.402	101.084±27.120	133.917±15.054
**Methylthiol**						
2MeSZR				2.839±1.009	10.657±3.531	11.058±3.425
2MeSZ					7.777±2.779	4.911±1.297
2MeSiPR					0.048±0.048	0.157±0.128
2MeSiP						

Values are means ± standard error (SE) (n = 2–15) at specific days post infection (dpi). Empty cells indicate values of zero for those analytes. In cases where an analyte was detected in only one sample the presented mean concentration is equivalent to the SE.

**Table 4:** Cytokinin concentrations (pmol g ^-1^ FW) in *U*. *maydis* solopathogen infected *Z*. *mays* cob tissue, during the *U*. *maydis*- *Z*. *mays* infection time course.

	Solopathogen (SG200)					
dpi	10	13	16	20	24	28
n	2	3	4	5	5	6
**Free base**						
iP						0.681±0.681
DHZ						
*trans*Z					1.618±1.177	1.210±1.210
*cis*Z					0.675±0.418	0.606±0.606
**Riboside**						
iPR	6.639±1.205	5.473±2.527	5.266±2.290	1.125±0.729	2.989±1.867	4.231±2.730
DHZR	1.298±0.001	0.512±0.140	0.402±0.159			
*trans*ZR	0.456±0.456	2.041±0.780	1.192±0.412	1.239±0.626	0.290±0.165	0.381±0.206
*cis*ZR	4.737±0.818	6.577±1.506	9.766±1.614	6.625±0.589	17.296±7.172	43.776±14.370
**Nucleotide**						
iPRP		5.753±1.152	12.449±4.122	2.996±1.284	1.746±1.075	1.555±1.049
DHZRP					1.070±1.070	3.553±2.248
*trans*ZRP	2.150±0.960	6.472±2.959	4.638±2.185	6.085±3.121	0.590±0.457	0.977±0.902
*cis*ZRP	9.552±4.151	13.696±5.122	40.350±9.122	40.066±10.466	36.132±10.872	49.930±8.874
**Glucoside**						
iP7G						
iP9G						
DHZOG						
DHZROG	4.965±4.965	35.314±4.853	53.615±10.033	51.055±6.515	22.559±3.466	12.849±4.752
DHZ9G						
*trans*ZOG						
*trans*ZROG						
*trans*Z9G	37.648±34.977	5.543±3.849	6.320±2.562	2.803±0.801	5.597±1.744	7.973±1.497
*cis*ZOG						
*cis*ZROG	115.323±12.890	240.574±12.735	284.930±41.237	225.570±23.700	179.474±60.483	109.364±18.227
*cis*Z9G	31.344±7.821	60.714±19.536	58.718±5.551	81.739±21.534	168.372±26.592	172.959±14.869
**Methylthiol**						
2MeSZR				0.397±0.257	2.275±0.987	4.468±1.565
2MeSZ					1.104±0.816	
2MeSiPR						
2MeSiP						

Values are means ± standard error (SE) (n = 2–15) at specific days post infection (dpi). Empty cells indicate values of zero for those analytes. In cases where an analyte was detected in only one sample the presented mean concentration is equivalent to the SE.

CK glucosides represented 93% (day 10) to 99% (days 13, 20 and 28) of the total CKs found in control tissue (Table 2). During dikaryon infection, glucosides represented the main CK type during early stages of infection at 81% and 82% at days 10 and 13. The relative amount of glucosides decreased at day 16 (65%) and was lowest at day 28 (47%) (Table 3). Methylthiol CKs (2MeSCKs) represented a small percentage of the total CK levels, and were present only in later stages of infected tissue, starting at day 20 (Tables 3 and 4). FBRNT CKs increased during dikaryon infection, representing over 50% of all CKs during later stages of infection (days 24, 28). During solopathogenic infection glucosides represented 73%-89% of the total CK level, whereas FBRNT CKs represented less than 17% of the total CKs detected during the time course, differing from the increased representation of FBRNT CKs noted in the dikaryon infection (days 10–24; Table 4). The following sections describe the dynamics of each CK grouping.

#### CK glucosides

**Fig 4:**
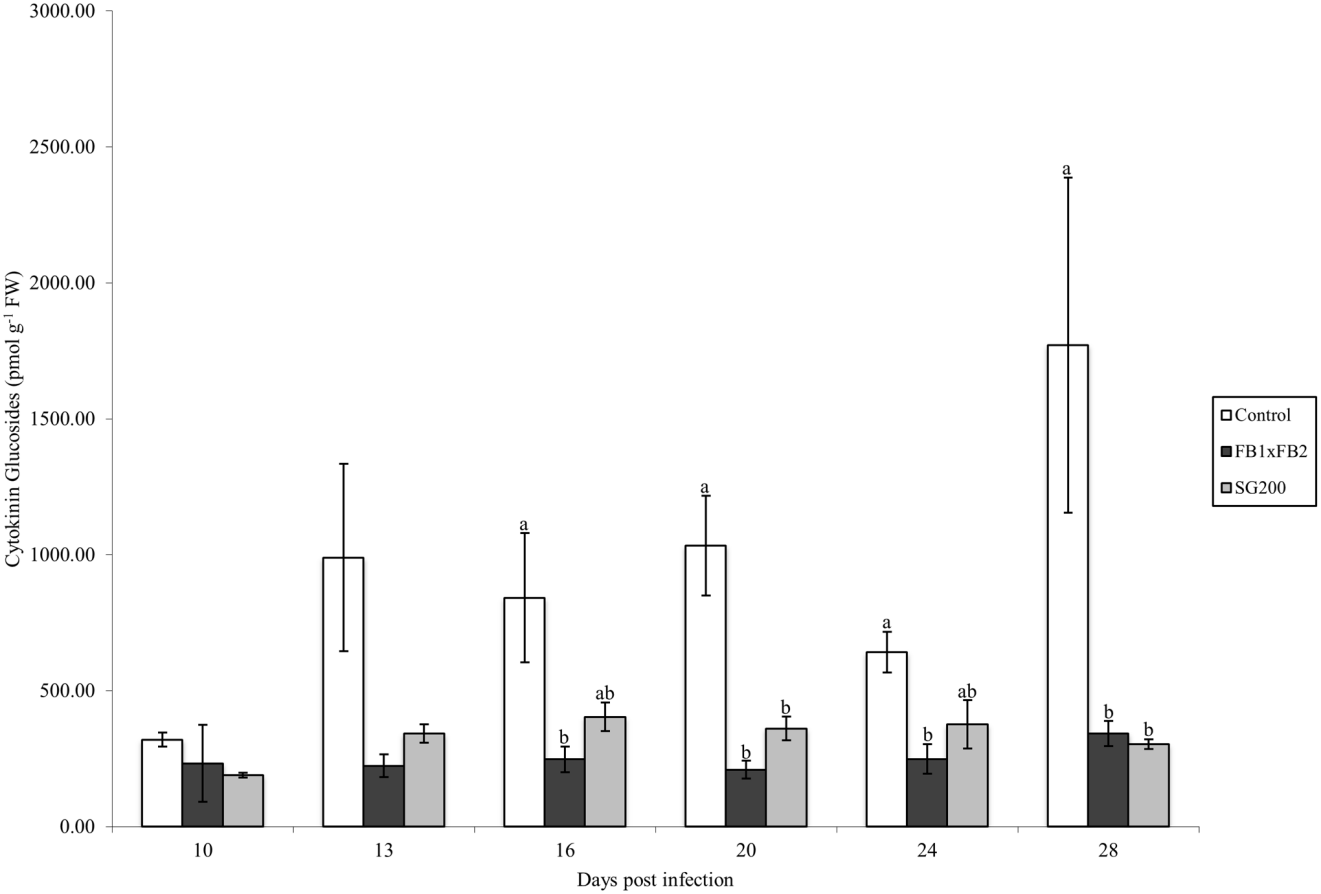
Total CK glucoside concentration (pmol g ^-1^ FW) for control, dikaryon and solopathogen injected cob tissue at specific days post infection (dpi). n = 2–15, error bars represent standard error (SE). Letters denote significant differences within time points (ANOVA and Tukey-Kramer post hoc analysis, *p*<0.05).

Total glucosides were compared between all treatments at each time point (Fig 4), with the control tissue maintaining the highest level of total glucosides throughout the time course. Total glucoside levels were significantly different between the control tissue and dikaryon tissue on days 16, 20, 24 and 28 (Tukey-Kramer *p*<0.05), and differences between the control tissue and solopathogen tissue were also noted at days 20 and 28 (Tukey-Kramer *p*<0.05).

Glucosides were further separated, based on the location of glucosylation, into *O-*glucosides (DHZOG, DHZROG, *trans*ZOG, *trans*ZROG, *cis*ZOG, *cis*ZROG) and *N-*glucosides (DHZ9G, *trans*Z9G, *cis*Z9G; Fig 3c), which revealed distinct profile changes within the tissues examined. *O-*glucosides can be readily cleaved by β-glucosidase and act as stable storage forms of CKs, whereas *N-*glucosides are thought to be irreversibly modified or inactive, as they are not cleaved by β-glucosidase [13,34]. Control tissue had higher levels of *N-*glucosides (>65% of total glucosides), specifically *trans*Z9G and *cis*Z9G throughout most of the time course (Table 2). Whereas infected tissue showed a reduction in *N*-glucoside forms, particularly *trans*Z9G and *cis*Z9G, relative to the control tissue, while having a higher representation of *O*-glucoside forms during days 10–20 (Tables 2, 3 and 4). Within infected tissue *cis*Z9G and *cis*ZROG represented the majority of detected glucosides (Tables 3 and 4).

#### Methylthiol CKs

**Fig 5:**
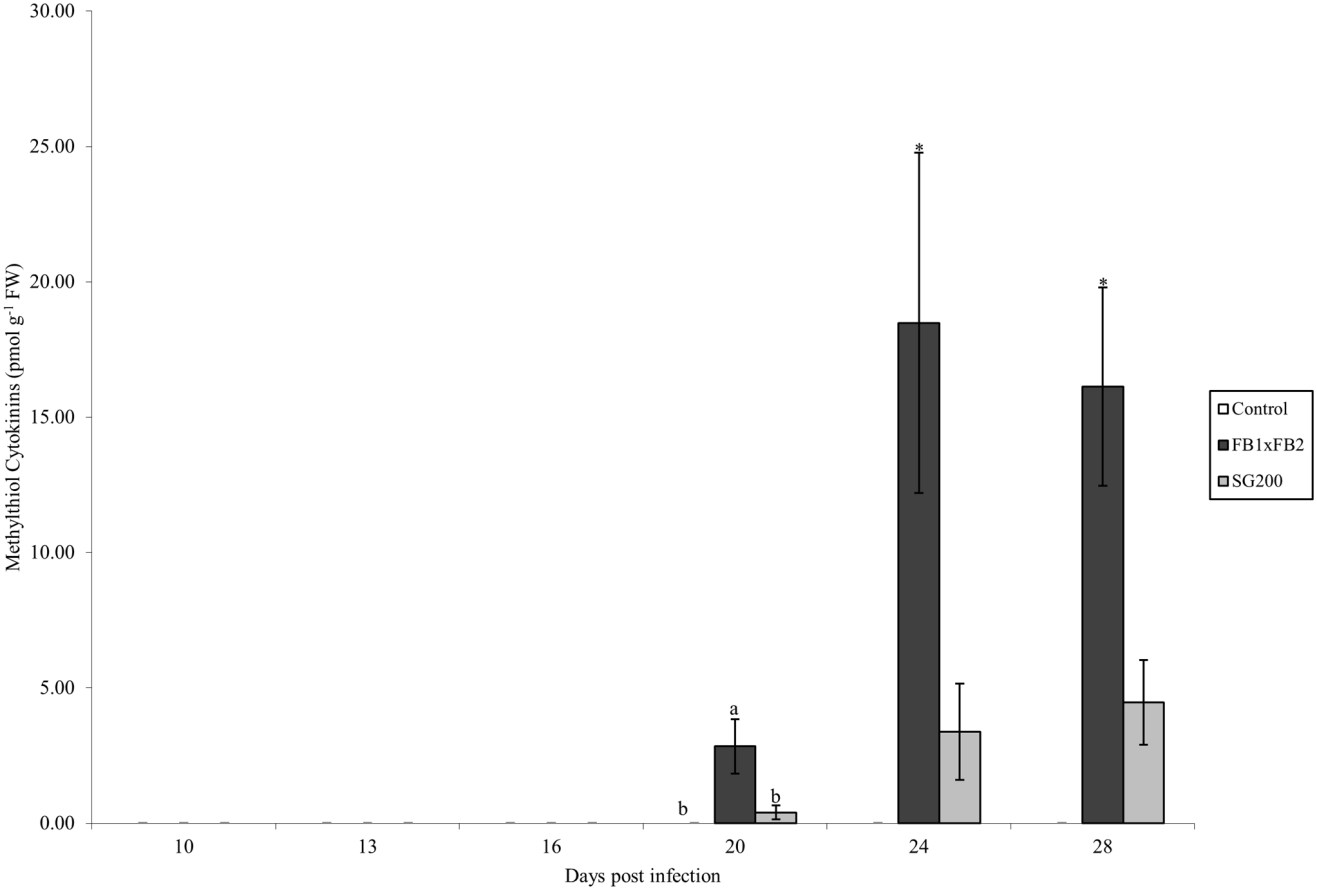
Total methylthiol CK concentration (pmol g ^-1^ FW) for control, dikaryon and solopathogen injected cob tissue at specific days post infection (dpi). n = 2–15, error bars represent standard error (SE). Letters denote significant differences within time points (ANOVA and Tukey-Kramer post hoc analysis, *p*<0.05). Asterisks (*) denote significant differences between the dikaryon and solopathogen infection within time points (two tailed Student *t*-test assuming unequal variance *p*<0.05).

Methylthiol CKs (2MeSCKs) were analyzed in the control, dikaryon and solopathogenic tissue (Fig 5). 2MeSCKs were not detected in control tissue at any time point; however they were detected in infected dikaryon and solopathogenic tissue beginning at day 20 (Fig 5). Total 2MeSCK levels increased after their initial detection at day 20 in both dikaryon and solopathogenic tissue. Significant differences were detected between the dikaryon tissue, and other tissues at day 20 (Tukey-Kramer *p*<0.05). Differences between the dikaryon and solopathogen infections were detected at day 24 and 28 (two tailed Student *t*-test assuming unequal variance *p*<0.05). Total 2MeSCKs in the dikaryon tissue ranged from 2.84 pmol g^-1^ FW at day 20 to >16 pmol g^-1^ FW for days 24 and 28, whereas solopathogenic tissue maintained low levels <4.5 pmol g^-1^ FW throughout the time course. 2MeSZR was the abundant 2MeSCK present in the infected tissue (Tables 3 and 4).

#### FBRNT CKs

**Fig 6:**
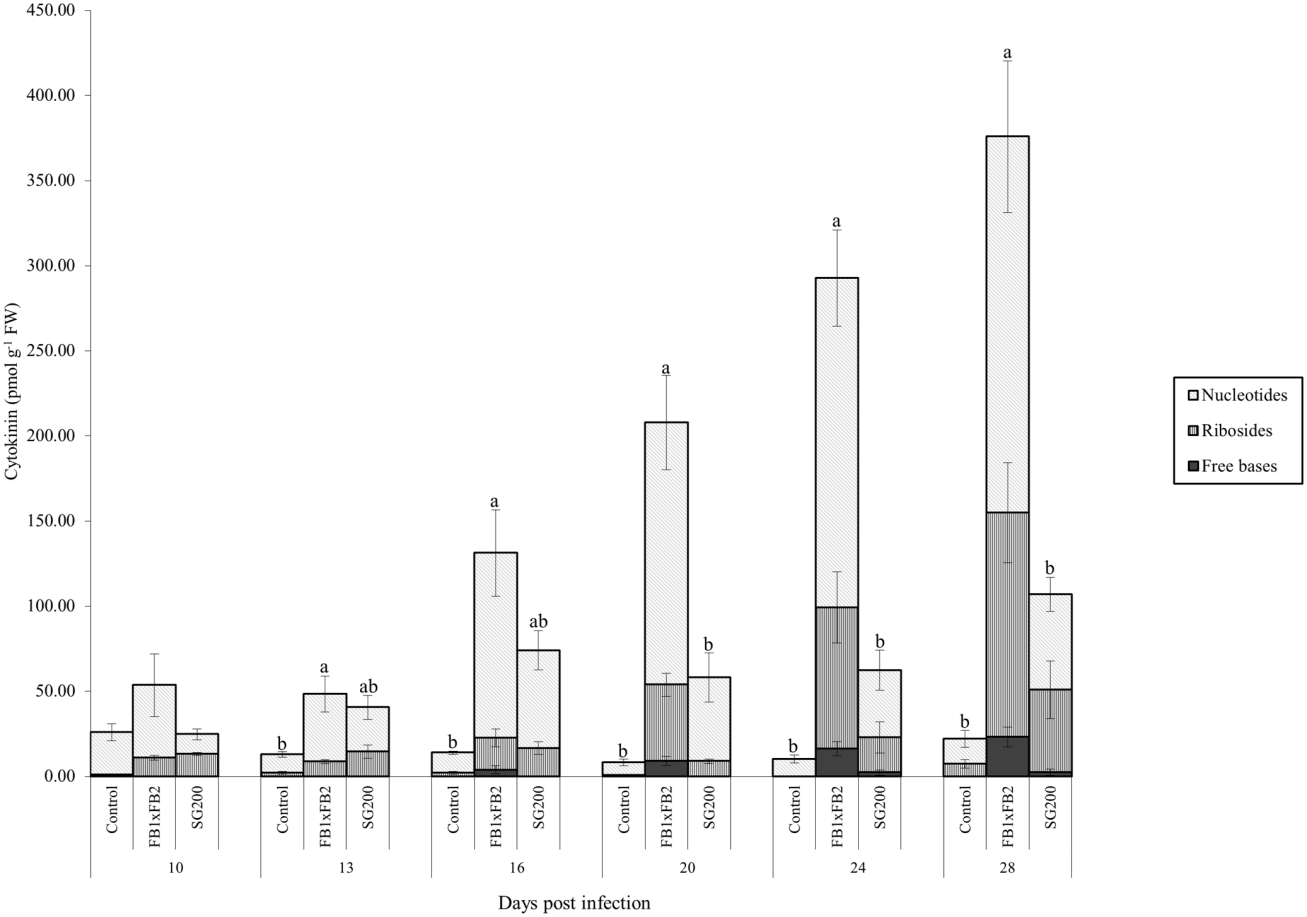
Total FBRNT CK concentration (pmol g ^-1^ FW) for control, dikaryon and solopathogen injected cob tissue at specific days post infection (dpi). Total FBRNT is subdivided into the Freebase, Riboside, and Nucleotide forms to show the representation of each form. n = 2–15, error bars represent standard error (SE). Letters denote significant differences within time points (ANOVA and Tukey-Kramer post hoc analysis, *p*<0.05).

Total free base, riboside and nucleotide (FBRNT) CKs varied among treatments throughout the time course. Specifically, in the control tissue, total FBRNT CKs maintained a concentration between 8 pmol g^-1^ FW and 26 pmol g^-1^ FW (Fig 6). CK levels were elevated in infected tissue relative to the control as the infection time course progressed. Dikaryon infected tissue had FBRNT levels significantly higher than control tissue at days 13–28 (Tukey-Kramer *p*<0.05), and significantly higher from solopathogen tissue at days 20–28 (Tukey-Kramer *p*<0.05) (Fig 6). The major FBRNT CKs found in the tissue samples were the nucleotide forms, followed by the ribosides, with low representation of the free base form across all treatments and time points (Fig 6). Further dividing FBRNTs into FBR forms, significant differences were detected at day 10 and 16 between the control and infected tissues (Tukey-Kramer *p*<0.05). Differences were also detected at day 20 between the dikaryon and other tissues (Tukey-Kramer *p*<0.05). While relatively low in total CKs, *cis*ZRP represented the major FBRNT CK in control tissue (>50%) for all time points with the exception of day 28 (Table 2). Infected tissue showed an accumulation of *cis*ZRP and *cis*ZR during the course of infection, with a 27-fold increase in *cis*ZR levels in the dikaryon infection and a 9-fold increase in *cis*ZR levels for the solopathogenic infection between days 10 and 28 (Tables 3 and 4). *cis*Z-isomer CK types constituted the majority of the total FBRNT CK pool in infected tissue as time progressed. iPCKs were found only in infected tissue starting at day 10 (Tables 2, 3 and 4) and increased in the dikaryon infection during the time course (Tables 3 and 4).

## Discussion

During host pathogen interactions CKs aid in the creation of nutrient sinks, as well as the formation of green islands or galls, whereas abscisic acid has been conflictingly implicated in increased host susceptibility as well as host resistance [4,5,35,36]. During *U*. *maydis* infection the formation of characteristic tumours is thought to be initiated by the release of fungal effectors whereas tumour expansion is likely dependant on plant hormones [18,37]. The knowledge that *U*. *maydis* produces phytohormones and that it induces different host responses in different tissues suggested the need to examine CK and ABA levels in infected cobs [23,38,39]. Tumour tissue collected at specific time points during dikaryon and solopathogen infection revealed slightly elevated levels of ABA, reduced CK glucosides, presence of 2MeSCKs, and dramatic increases in specific FBRNT CKs. These distinct alterations in the phytohormone profile indicated that *U*. *maydis* uses phytohormone manipulation as one of its approaches to modulate the plant host’s physiology.

### *U*. *maydis* cob infection leads to phenotypic differences between two strains and among tissue collections

Dikaryon and solopathogen infections differed in pathogenic development and phytohormone levels. Tumour formation progressed similar to that outlined in Pataky and Snetselaar [28] with slightly reduced virulence, or less pronounced visual symptoms in the solopathogen infections relative to the dikaryon infection at early time points (days 10–16).

This variability in virulence between the dikaryon and other *U*. *maydis* strains is consistent with previous observations by Babu et al. [40] (dikaryon vs. diploid) and the Saville Lab (S1 Fig, dikaryon vs. solopathogen) in corn seedling infections. The differences in phytohormone levels noted in the current study provide a possible link between strain type and virulence level in the *U*. *maydis*- *Z*.*mays* interaction.

Virulence differences have been noted between strains, yet the severity of *U*. *maydis* cob tumour formation can also be influenced by the time of inoculation and pollination [26]. In order to control for pollination, corn was detasseled in the current study. Snetselaar et al. [25] noted that when cobs were shielded from pollen they were more susceptible to *U*. *maydis* infection. Pollination may act to protect ovaries from infection due to the formation of an abscission zone at the base of the silk preventing multiple pollen tubes from reaching the ovary and inhibiting *U*. *maydis* entrance into the ovary [25]. Furthermore, fertilized seeds yield a very distinct CK profile from unfertilized ovules [41]. For these reasons careful visual inspection of the cob was done in order to exclude potentially fertilized seeds from the control analysis, as unfertilized tissue more accurately represented the tissue that would be infected by *U*. *maydis*. Moreover, consistent sampling times-of-day were used to control for potential fluxes in CK diurnal levels [42].

### Hormone profiling

#### ABA levels are elevated in infected tissue

ABA plays a key role in plant development including seed dormancy and response to abiotic and biotic stressors [5,7,8,43]. Increased levels of ABA have been associated with elevated susceptibility of plants to pathogen attack [5](reviewed in [44]); however callose deposition stimulated by ABA can also provide a degree of resistance [7]. The role of ABA in host-pathogen relations is influenced by the infection strategy of the pathogen and the infection stage at which it is triggered [36]. Hence ABA’s role in pathogen infection is finely balanced. In the current study the increase in ABA at earlier time points (day 10, Fig 2) in *U*. *maydis* disease development suggests a possible role for ABA in initiating host susceptibility and tumour expansion. Increased ABA levels due to exogenous application or fungal production have resulted in increased susceptibility of the tomato plant to *Botrytis cinerea* infection [5], and rice to *Magnaporthe grisea* infection [45]. *U*. *maydis* is capable of producing ABA, and ABA levels were elevated at 14 dpi during corn seedling infection [23]. This increased susceptibility associated with ABA may also be due to the complex interaction and cross-talk between many phytohormones [46]. Studies have detected an antagonistic interaction between ABA and salicylic acid (SA) which influences host susceptibility. SA production is an important defense response strategy for plants and is commonly associated with defense against biotrophic pathogens [46]. Pre-treatment of plants with ABA results in the suppression of the SA pathway. SA suppression has been detected during the interaction between *Magnaporthe grisea* and rice [45], *Arabidopsis thaliana* and *Pseudomonas syringae* pv.tomato [47], *Xanthomonas oryzae* pv. *oryzae* and rice [46] among others resulting in increased host susceptibility. Suppression of plant SA appears to play a minor role in *U*. *maydis* virulence [48,49] and deletion of an *U*. *maydis* SA-degrading enzyme, salicylate hydroxylase, does not appear to impact virulence during corn seedling infection [50]. Many complex interactions occur between phytohormone systems; manipulation of these by the pathogen can greatly influence plant immunity [46]. The proposed initial suppression of host defenses through elevated ABA levels, as seen in the current study, is consistent with development within the *U*. *maydis*–*Zea mays* pathosystem.

During the dikaryon infection ABA levels were elevated above control levels at day 13 and quickly decreased by day 16. This pattern is modified in the solopathogen infection as ABA levels were >2 fold higher than the dikaryon at day 16 and were higher than the dikaryon infection from days 13–20. The higher level of ABA in the solopathogen infection may play a role in the virulence reduction noted during the time course. Elevated levels of ABA are known to result in decreased cell division in normal maize seed development and can also trigger other plant hormone defense responses when produced later on in the infection process (proliferation stage) [36,51]. The mock-infected control follows a different pattern for ABA accumulation when compared to the infected tissue. In control tissue, ABA levels gradually increased. During normal seed development, increases in ABA facilitate the maintenance of seed dormancy [52] and two ABA peaks occur during early and late seed maturation stages [53], ABA levels are not typically measured in unfertilized ovules. In this study, the increased ABA level detected in unfertilized ovules was likely due to the natural dessication of the cob.

In the current system ABA may allow for increased susceptibility of the host to *U*. *maydis* infection initially, but past a certain point in *U*. *maydis* development may actually result in decreased cell division and lower virulence.

#### CKs in tumour formation

During normal seed development cytokinins act to stimulate cell division and lead to increased organ size and enhanced sink strength (reviewed in [54]). However, during infection, *U*. *maydis* does not simply grow within the constraints set for a developing seed, but manipulates and reprograms the already existing sink tissue in order to complete its lifecycle.

In the current study total CKs decreased upon *U*. *maydis* infection, due to an overall reduction in CK glucosides (Tables 2, 3 and 4). A similar decrease in total CKs was also reported by Behr et al. [55] during *Colletotrichum graminicola* infection of maize leaves, and was likely due to considerable decreases in *O*-glucosides. However *U*. *maydis* infection, while resulting in the decrease of CK glucosides, results in the accumulation of more active CK forms and their precursors. By subdividing CKs into groups, as per the current study, changes in the precursor, active, storage and sometimes overlooked CKs (i.e. 2MeSCKs) can more clearly be seen. All of these CKs appear to play an important role in this pathosystem.

#### CK glucosides

CK glucoside levels decreased considerably during infection, particularly the *trans*Z and DHZ forms, which all but disappeared. *cis*Z glucoside forms remained relatively high in infected tissue, suggesting that glucosylation was limited to *cis*-isomers in infected tissue. Notably, the levels of *N-*glucosides were reduced in infected tissue when compared to the mock-infected control. The reduced glucoside levels in infected tissue may be due to the fungus acting to inhibit the plant’s glucosylation activity by potentially targeting *Zea mays* N9-glucosyl transferase (ZmCNGT), responsible for *N-*glucosylation of CKs, or other zeatin-*O-*glucosyltransferase genes [56,57]. Changes in CK glucoside balance appear to be highly tissue and pathogen specific. *C*. *graminicola* infection of maize leaves results in significant decreases in *cis*ZOG following infection [55]; whereas in the current study the *N-*glucosides: *trans*Z9G and *cis*Z9G were greatly reduced upon cob infection by *U*. *maydis*.

Another way in which glucoside balance can be altered is through the activation of β-glucosidases [58], which catalyze the deglycosylation of *O-*glucosides [34]. β-glucosidase activity has been detected in other fungi [3], and β-glucosidase genes have been identified in *U*. *maydis* with elevated expression levels during corn seedling infection (5 and 13 days post infection; [15]). One such gene, *UMAG_00446*, is characterized as a probable β-glucosidase, the enzyme commission number (EC) for UMAG_00446 (EC 3.2.1.21) is the same as the identified EC for β-glucosidase listed in Spichal [13]. This consistency between UMAG_00446 and listed β-glucosidases suggests that this *U*. *maydis* enzyme could function to convert the more abundant CK *O*-glucosides to active CK free bases or ribosides during the course of infection [6,15]. In the current study *trans*Z derived as well as *cis*ZR CKs increased in dikaryon infected tissue as time progressed (Table 3). In this case, the fungus may be hijacking the already existing environs of the plant to increase its sink strength through the release of active CKs.

#### 2MeSCKs

The function of methylthiol CKs (2MeSCKs) is not well understood and very little is known regarding their importance in plant systems. Recent reports have detected 2MeSCKs in various basidiomycete forest fungi [14], and have also highlighted their potential importance in plant-insect-microbe interactions [59]. *U*. *maydis* is not known to produce 2MeSCKs separate from the plant yet 2MeSCKs have been found as components of the tRNA of all organisms with the exception of Archaea [13,60,61]. In the current study, 2MeSZR and 2MeSZ accumulated in *U*. *maydis* infected tissue during the later stages of infection but were not detected in the control tissue. Little is known about tRNA accumulation/degradation during end stage *U*. *maydis* infection. The potential for tRNA accumulation off set by degradation may influence the level of 2MeSCKs detected during the current infection time course. 2MeSZ and 2MeSZR were the more predominant 2MeSCKs detected in the current study, this parallels the findings of Morrison et al. [14]. Furthermore, pathogenic strains of *Rhodococcus fascians* contain the same spectrum of CKs as their nonpathogenic counterpart yet with higher levels of 2MeS*cis*Z, iP and *cis*Z, of which 2MeS*cis*Z and *cis*Z were found to accumulate in infected *Arabidopsis thaliana* tissue [62]. Little is known about the bioactivity of 2MeSCKs, but the authors suggested that 2MeSZ may be less cytotoxic than other classic cytokinins and therefore this may account for its accumulation during infection [62]. The low but consistent presence of 2MeSCKs may be important in eliciting plant responses during disease development [59]. The similar accumulation of 2MeSCKs in *U*. *maydis* infected tissue may be functionally based in the fact that they are less cytotoxic to the plant or they may also evade attempts by the host to balance CKs through the use of cytokinin oxidase.

Cytokinin oxidase acts to permanently degrade CKs; however, 2MeSZ and *cis*Z, were found to be poor substrates for 3 apoplastic cytokinin oxidases, permitting their accumulation in tissue [62]. Although fungi are capable of producing 2MeSCKs [14] it may be that host-stimulated or fungal-supplied 2MeSCKs, during infection, influence the host cells and permits the continued proliferation of tissue around the site of infection. In the current study the presence of 2MeSCKs in infected tissue may indicate a fungal stimulated origin for these CKs and their importance in promoting tissue proliferation.

#### FBRNTs

FBRNTs include free base, riboside and nucleotides which respectively represent the active, transport and precursor forms, of these CKs [34,55,63]. In this study they have been grouped together to reflect the importance of the NT-R-FB pathway in eliciting classic CK responses in plants. Fertilized seeds have a peak in CK levels 6 days after pollination, which is earlier than unfertilized ovules. Fertilized seeds also have CK levels 60 fold higher than unfertilized ovules [41,52]. *U*. *maydis* tissue does not mirror the early CK peak seen in fertilized seeds, suggesting that it does not simply mimic a fertilized seed, but instead reprograms the host tissue. FBRNTs, notably *cis*ZRP and *cis*ZR, increased dramatically in dikaryon and solopathogen infected cob tissue relative to the control starting at day 16 (Fig 6). This similar *cis*ZCK accumulation has been detected during maize leaf infection by *C*. *graminicola*, corn seedling infection by *U*. *maydis*, and *Arabidopsis thaliana* infection by the bacterium *Rhodococcus fascians* [23,55,62]. CK accumulation is likely dependent on host substrate specificities [62]. The abundance of *cis*Z type CKs in maize [64], cytokinin receptors that are responsive to *cis*Z CKs [65], and zeatin-*O-*glucosyltransferase genes specific to *cis*ZCKs [56,57], suggests that maize has the capacity to respond to the accumulation of *cis*ZCKs during *U*. *maydis* infection.

CK levels did not change dramatically in control tissue during the time course; which supports the approach of this study to only use unfertilized ovules. The high levels of glucosides in the unfertilized control tissue in the current study may reflect a counter-balance to the low levels of FBRNT CKs detected. The accumulation of FBRNTs in infected tissue may also be due to the inhibition of CK degradation which is primarily driven by cytokinin oxidase (CKX) in most plant systems [66]. CKX removes the N^6^-substituted isoprene chain of CKs or their ribonucleosides to produce adenine and the corresponding aldehyde, thus removing any CK activity (reviewed in [66]). Brefort et al. [67] found that the *Zea mays* cytokinin oxidase 3 gene is up-regulated following infection by *U*. *maydis* strains in which an effector gene cluster has been deleted; suggesting that during normal infection these effectors act to suppress CK oxidase production. Microarray data from *U*. *maydis* infected corn seedlings [48] noted a >3 fold increase in the *Zea mays* CK receptor: AHK4 histidine kinase receptor, starting at 4 dpi as well as a decrease in a *Zea mays* potential glycosyltransferase at 2 dpi following *U*. *maydis* infection of corn seedlings. These changes in host gene expression profiles suggest that *U*. *maydis* is capable of manipulating the host’s CK signaling and storage pathways. Furthermore, during normal *Z*. *mays* development CKX levels are higher in the embryo, in order to prevent precocious germination [52]. *U*. *maydis* appears to take over the role of the embryo, resulting in the collapse of the ovule inside the ovary and a hollow appearance of tumours [25]. This targeted suppression would effectively hamper the plants ability to control CK balance within the seed, and lead to specific (*cis*-isomer) CK accumulation; this is supported by the current study’s phytohormone infection profiles.

## Conclusions

This study found that *U*. *maydis* infection specifically alters the CK balance within *Zea mays*, with specific reduction of CK glucosides and increases to *cis*ZCKs, iPCKs and 2MeSCKs. CK accumulation is more dramatic in the dikaryon infection and may account for the greater tumour manifestation seen during this infection. The increased CK levels may be working to promote sink development, distribution of nutrients and increased cell division, which may effectively reprogram the host tissue resulting in larger tumours. The changes in ABA were not as clear as has been seen previously in *U*. *maydis* infected corn seedlings [23]. However, ABA accumulation and maintenance during early and later cob infection stages likely requires a delicate balance. Further study must be done in order to determine if the higher maintained levels of ABA starting at day 16 (in solopathogen infections) can result in heightened plant response and reduced virulence.

CK homeostasis requires a direct balance of the rate of import, biosynthesis, inactivation and degradation [66]. Based on the CK profiles in this study it is hypothesized that the accumulation of specific CKs (cytokinin-mix strategy) [59], induction of fungal β-glucosidase, as well as the manipulation of cytokinin oxidase and CK-glucosylation activity in the plant, play a role in the resulting CK accumulation and tumour development in infected tissues. *U*. *maydis*, through various factors, including the modulation of phytohormones, effectively hijacks and reprograms the host tissue. CK biosynthesis genes have been identified in *U*. *maydis* and are currently under investigation [14]. Examination of CK biosynthesis genes in other fungi including the ergot fungus *Claviceps purpurea*, indicated that deletion of an isopentenyltransferase fused ‘*lonely guy*’ gene (*ipt-log*) and a p450 monooxygenase gene (involved in the hydroxylation of the isopentenyl side chain) specifically impacts *trans*Z production; however, it has no impact on pathogenesis during infection of rye plants [68]. CKs secreted by fungi also impact other phytohormone pathways; for example, during interactions between *Magnaporthe oryzae* and rice, CK secretion by the fungus enhances the SA defense response of the rice plant. This highlights the fact that a fine balance of multiple phytohormone systems is necessary for successful pathogen establishment [69]. We postulate that the ability of *U*. *maydis* to synthesize CKs [23] and specifically manipulate *cis*Z CK levels, influences the establishment and pathogenicity of this interaction. Furthermore, fungal CK accumulation likely stimulates CK signaling and metabolism genes within the host plant as seen in other systems ([68,69] and others). All of these factors play a role in *U*. *maydis* pathogenicity, and further identify potential target genes that may be important to examine within the context of *U*. *maydis* and *Z*. *mays* CK metabolism.

## Supporting Information

S1 Fig Pathogenesis assay for *U*. *maydis* infected corn seedlings at 14 days post infection. The dikaryon (FB1x FB2) and solopathogen (SG200) strains of *U*. *maydis* were injected into seven day old corn seedlings and pathogenesis scored using the disease symptoms presented in the legend. The percentage of symptom formation is indicated for each treatment. A non-parametric Mann-Whitney *U* test was conducted to assess statistical significance (*p*<0.05). Statistical significance is indicated by an asterisk (*). n equals total sample size. Each disease symptom was assigned a numerical value; the average of this is represented by the value associated with the disease index (D.I.).
(TIF)
