# Supplementary figures and images for: Phytohormone Involvement in the Ustilago maydis– Zea mays Pathosystem: Relationships between Abscisic Acid and Cytokinin Levels and Strain Virulence in Infected Cob Tissue
Source: PLoS One. 2015 Jun 24;10(6):e0130945. doi: 10.1371/journal.pone.0130945 (PMC4479884; doi:10.1371/journal.pone.0130945)

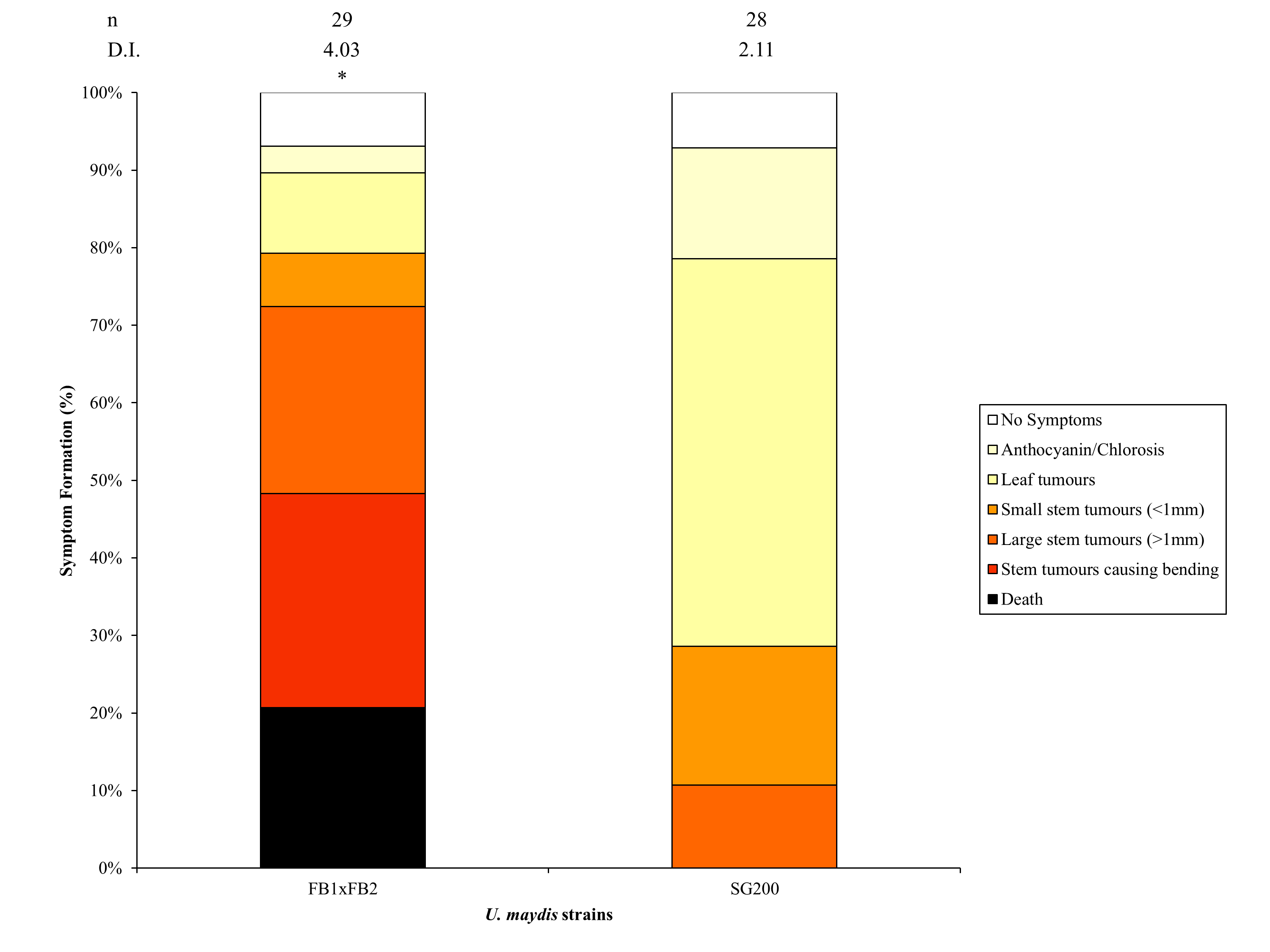

Supplement: S1 Fig — The dikaryon (FB1x FB2) and solopathogen (SG200) strains of U. maydis were injected into seven day old corn seedlings and pathogenesis scored using the disease symptoms presented in the legend. The percentage of symptom formation is indicated for each treatment. A non-parametric Mann-Whitney U test was conducted to assess statistical significance (p<0.05). Statistical significance is indicated by an asterisk (*). n equals total sample size. Each disease symptom was assigned a numerical value; the average of this is represented by the value associated with the disease index (D.I.). (TIF) [file pone.0130945.s001.tif]
